# Insight into Apert Syndrome: Reporting on Six Patients and Increasing Awareness

**DOI:** 10.1007/s12035-025-04902-9

**Published:** 2025-04-22

**Authors:** Hala T. El-Bassyouni, Ghada Y. El-Kamah, Hanan H. Afifi, Mohamed B. Taher, Doaa R. Soliman, Khaled Hamed, Mennat I. Mehrez, Khalda Sayed Amr

**Affiliations:** 1 Department of Clinical Genetics, National Research Centre, Cairo, Egypt; 2 Department of Pediatrics, Faculty of Medicine, Benha University, Benha, Egypt; 3 Department of Oro-Dental Genetics, National Research Centre, Cairo, Egypt; 4 Department of Molecular Genetics, National Research Centre, Cairo, Egypt

**Keywords:** Apert syndrome, Craniosynostosis, Dental anomalies, *FGFR2*, Syndactyly

## Abstract

Apert syndrome (AS) is a rare autosomal dominant disorder characterized by various congenital malformations. In this study, we aimed to explore the clinical presentation of Apert syndrome to enhance awareness among multidisciplinary healthcare providers regarding its differential diagnosis through the phenotype/genotype characterization of six Egyptian patients with AS. We examined six patients with Apert syndrome: four females and two males (2:1), aged 3 to 7 years. Clinical examination, along with pedigree analysis, was followed by DNA extraction from the patients’ and their parents’ peripheral blood leukocytes for genomic screening of *FGFR2* gene variations. Key findings in all patients included craniosynostosis and distinctive facial features such as midface hypoplasia, exophthalmos, hypertelorism, a beaked nose, a prominent forehead, and an underdeveloped upper jaw, along with syndactyly of the hands and feet. We identified oral anomalies such as cleft palate, bifid uvula, impacted teeth, delayed eruption, supernumerary teeth, and thick gingiva. Pathogenic variants of the *FGFR2* gene were characterized in all six patients. This report presents the largest cohort of Apert syndrome among Egyptian patients. Raising awareness about AS, especially among various interdisciplinary teams, is essential for managing this rare condition and is crucial for accurate diagnosis and timely medical and surgical intervention. Proper diagnosis and genetic counseling are necessary for improving survival and preventing the recurrence of complications.

## Introduction

Apert syndrome (AS) (MIM #101,200), also known as acrocephalosyndactyly type 1, affects multiple organs and manifests in various clinical forms, influencing skeletal, cutaneous, respiratory, and visceral characteristics [1]. This rare genetic disorder has a global incidence of 1 in 65,000, with only 1 in 160,000 individuals surviving past infancy. AS makes up 4.5% of cases of craniosynostosis and is caused by pathogenic variants of the FGFR2 gene [2, 3]. Several craniosynostosis syndromes, including Crouzon, Apert, Pfeiffer, Antley-Bixler, Beare-Stevenson cutis gyrata, Jackson-Weiss, Bent bone dysplasia, and Seathre-Chotzen syndromes, arise from pathogenic variants in FGFR2 (MIM 176943) [4].

In the case of AS, patients exhibit craniofacial defects and malformations alongside craniosynostosis, including midface hypoplasia, exophthalmos, hypertelorism, a beaked nose, and syndactyly of the hands and feet [5, 6]. Additional skeletal features include reduced glenohumeral mobility, a short humerus, radiohumeral synostosis, pectus excavatum, an asymmetric chest wall, chest wall flattening, spina bifida, hemivertebrae, spinal fusions, scoliosis, lordosis, wide interpubic distance, genu valga, and osseous ankylosis at the knees [7]. Patients with AS may also experience polydactyly, intellectual impairment, obstructive sleep apnea, and hearing loss [8].

Cleft palates and bifid uvulas account for approximately 75% of AS cases; oral abnormalities are often observed in AS patients [9]. Intraoral features have been documented, such as impacted teeth, delayed eruption, ectopic eruption, supernumerary teeth, and thick gingiva [10]. Occlusal examinations also indicated the presence of an anterior and posterior open bite, as well as crossbite [11]. The *FGF* gene is crucial in regulating tooth development by controlling reciprocal epithelial and mesenchymal interactions [12]. The expression of the mutant receptor results in the disorganization of epidermal keratinocytes, leading to epidermal thickening [13].

Most patients with *FGFR2*-related conditions experience craniosynostosis involving both minor and major suture fusion. *FGFR2* is predominantly expressed in the cartilages of the cranial base and epiphyseal dysplasia, as well as in differentiating osteoblasts and osteoprogenitor cells of the chondrocyte lineage in Apert syndrome [14, 15]. Identifying *FGFR2* pathogenic variants in craniosynostosis highlights their vital role as signal transducer molecules during the normal development of cranial sutures [16]. The four recognized fibroblast growth factor receptors (FGFRs) are *FGFR1*, *FGFR2*, *FGFR3*, and *FGFR4*. Each *FGFR* comprises an extracellular domain, a transmembrane region, and a cytoplasmic tyrosine kinase domain. The FGFR2 protein consists of three immunoglobulin-like subdomains (IgI, IgII, and IgIII), which regulate the signaling of both FGF and *FGFR*, crucial for mesoderm formation, cell migration and growth, organ development, and bone growth [17, 18].

Significantly, little is understood about the abnormal molecular mechanisms of cell differentiation in AS, particularly regarding the activation of this process, which may be substantially influenced by the signaling pathways triggered by the *FGFR2* gene variant [19]. All patients with Apert syndrome had a spontaneous base substitution of variants originating from the father at one of the two most reported nucleotides, NM_000141.5(*FGFR2*): c.755 C > G or c.758 C > G in the *FGFR2* gene. Premeiotic cells in the male germline carrying these pathogenic variants possess a selection advantage due to these variants, even if detrimental to the child. The incidence of pathogenic variants also rises with paternal age [3].

The role of *FGFR2* in many craniosynostosis genetic syndromes presents a diagnostic challenge, particularly for non-geneticist members of multidisciplinary teams involved in AS management. We report on the clinical and molecular assessment of *FGFR2* in six Apert syndrome patients from six Egyptian families, highlighting the importance of raising awareness about the cardinal phenotype of AS and informing physicians about variability and genetic counseling decisions.

## Patients and Methods

Six patients (4 females and 2 males) were clinically diagnosed with AS based on the presence of its cardinal clinical manifestations. They were recruited from the Clinical Genetics Clinics at the Center of Medical Excellence, National Research Centre, following the approval of the Institutional Review Board of the NRC following the Helsinki Declaration. Informed consent was obtained from the parents of all patients who agreed to have their images and other data used for research and publication.

## Clinical Assessment

All patients were the offspring of nonconsanguineous marriages. Their ages ranged from 3 to 7, and the female-to-male ratio was 2:1. The paternal age ranged from 28 to 48.

Patients’ complete medical histories, including gender, age, primary complaint, pregnancy or postnatal problems, and history of current illness, were collected after all participants’ legal guardians provided written informed consent. Pedigree analysis for at least three generations highlighting consanguinity, paternal age, and family members suffering similar or different genetic abnormalities. Thorough clinical evaluation through a multidisciplinary team, including a clinical geneticist, a dental geneticist, and other specialties, through needed referrals. Radiologic and laboratory analyses were performed when indicated. Five milliliters of blood were obtained from patients and their parents in EDTA tubes for DNA extraction.

## Molecular Investigation

Targeted Sanger sequencing analysis of exon7 in the *FGFR2* gene was used for all studied patients and available parents using a DNA Mini Kit (Qiagen) following the manufacturer’s instructions, and quantification of the isolated DNA was performed by spectrometry in a NanoDrop 2000. We used the Agilent Sure Select Target Enrichment System procedure to produce a sequencing library from the genomic DNA of the patient. Using the MEM approach reads containing pathogenic variations were identified and prioritized before being aligned to the human genome reference sequence (hg19) using Burrows-Wheeler Aligner version 0.7.5 [20]. Version 1.93 of Picard (http://broadinstitute.github.io/picard/) and SAMTOOLS version 0.1.18 of GATK version 2.4–7 [21] were utilized for sorting and indexing SAM/BAM files, local realignment, and duplication marks. GATK (known single-nucleotide polymorphisms and indels from dbSNP137, Mills, and 1000 Genome Project gold-standard indels from b37 sites, as well as the 1000 Genome Project phase 1 indel from b37 sites) was used to identify variants from the targeted genes. Sequence variants were called using GATK’s Unified Genotyper and recalibrated using dbSNP137 to find pathogenic variants in the genes. Variant filtration, analysis, and ANNOVAR10 were used to annotate the variants.

## Sanger Sequencing Confirmation

Segregation analyses among parents of affected siblings and available family members were conducted using Sanger sequencing. Amplification of exon 7 IIIa of the *FGFR2* gene was performed using PCR with primers designed by Primer 3 (https://primer3.ut.ee/) to facilitate the detection of pathogenic variants. The primer sequence of *FGFR2*- F: CTTCCCGTATTCATCAGGT R: CATCCTCTCTCAACTCCAACAG. Following the manufacturer’s instructions, the PCR results were purified using an Exo-SAP PCR purification kit (Fermentas, Germany). Both directions were sequenced using the Big Dye Terminator v3.1 Cycle Sequencing Kit and examined on the ABI Prism 3500 Genetic Analyzer (Applied Biosystems).

Sequence variants were compared to those in the *FGFR2* gene variant database after matching with the appropriate wild-type sequences using BLAST (NCBI).

## Gene–Gene Interactions

The Gene–Gene Interaction analysis for the *FGFR2* gene was conducted using GeneMANIA (https://genemania.org/). GeneMANIA integrates multiple association networks to predict gene function in real-time. It employs a rapid heuristic algorithm based on ridge regression to merge various functional association networks, predicting gene function through label propagation within a single process-specific network. The analysis included data from genomics and proteomics sources processed into weighted interaction networks. These networks were standardized to enhance the accuracy of the predictions [22].

## Results

**Fig. 1:**
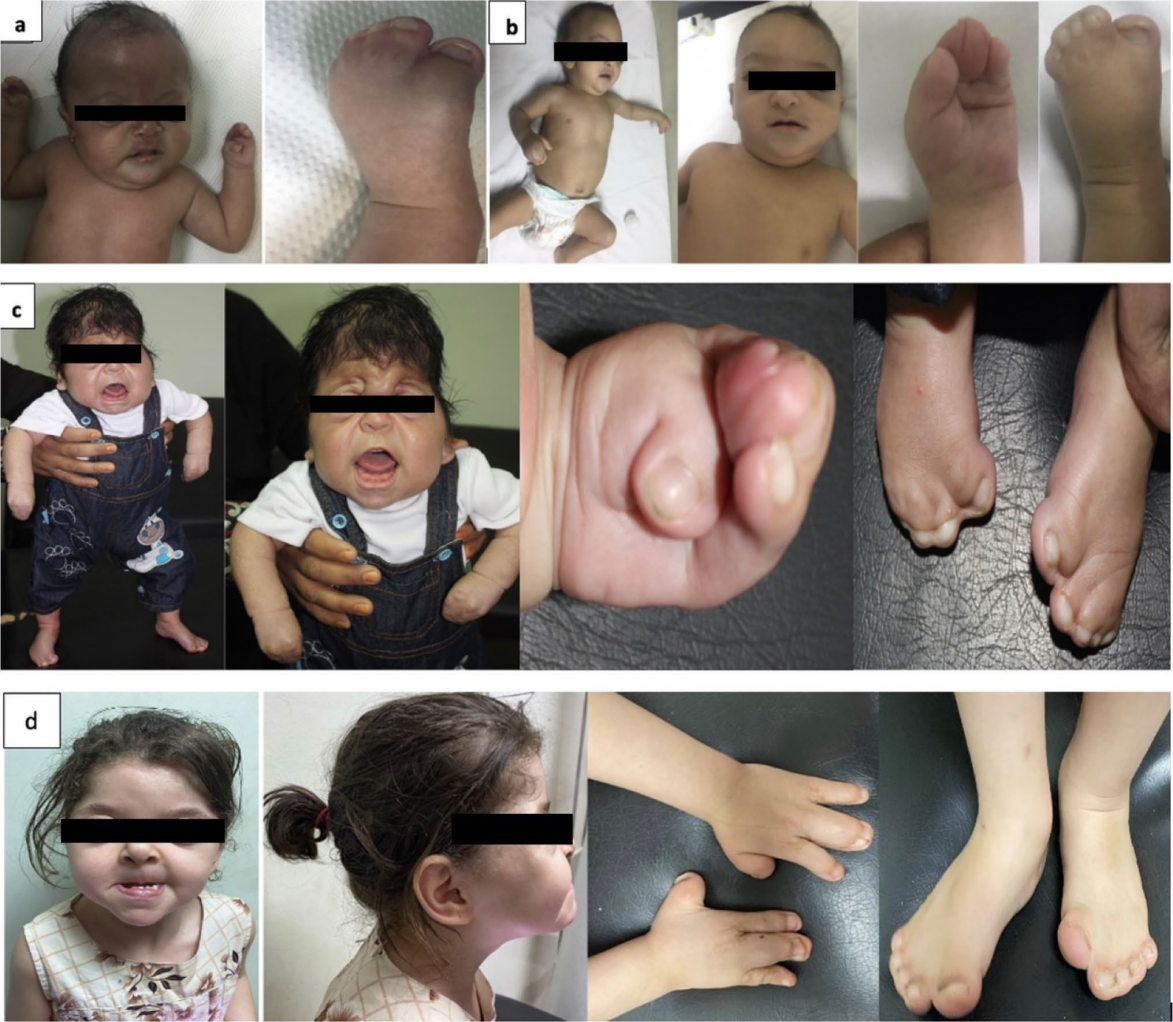
Clinical phenotype of patients: **a**: Patient 1 shows a broad forehead, frontal bossing, proptosis, depressed nasal bridge, long philtrum, syndactly, and broad foot. **b**: Patient 2 shows broad forehead, frontal bossing, proptosis, depressed nasal bridge, short philtrum, retrognathia, maxillary hypoplasia, syndactly, and broad hand and foot. **c**: Patient 4 shows depressed nasal bridge, hypertelorism, beaked nose, long philtrum, and syndactly of hand and feet. **d**: Patient 5 shows a broad forehead, proptosis, depressed nasal bridge, short philtrum, syndactly, and broad hands and feet. X-rays of the hand and feet shows bone syndactly

All patients presented with cardinal signs of AS, including craniosynostosis, depressed nasal bridge, proptosis, midface hypoplasia, and syndactyly of the hands and feet. Acrocephaly and frontal bossing were present in 5 patients (83.7%) (Table 1 and Fig. 1). Two patients (patients 4 and 6) had congenital heart disease (patent ductus arteriosus and patent foramen oval) (33.3%). All patients presented with normal MRI brain results; however, it is noteworthy that patient 5 displayed agenesis of the corpus callosum (Fig. 3). Skeletal anomalies characterized by broad hands and toes were observed in four patients, and one patient presented with bilateral talipes equinovarus (patient 3), who was also the only patient who had a cleft palate detected. Polydactyly was present in one patient (16.7%) (Table 1 and Fig. 1); none presented with sensorineural hearing loss. Moreover, dental anomalies such as cleft palate (66.7%), bifid uvula (50%), impacted teeth (33.3%), delayed eruption (33.3%), supernumerary teeth (16.7%), and thick gingiva (16.7%) were noted (Table 1).

**Table 1:** The clinical characteristics of AS patients

	Patient 1	Patient 2	Patient 3	Patient 4	Patient 5	Patient 6
Age/gender	4 y/F	3 y/M	7 y/F	7 y/M	4 y/F	3y/F
Consanguinity	-	-	-	-	-	-
Dysmorphic features	Broad forehead, depressed nasal bridge, long philtrum	Depressed nasal bridge, short philtrum, retrognathia, maxillary hypoplasia	Depressed nasal bridge, hypertelorism, beaked nose, long philtrum	Depressed nasal bridge, absent eyebrows, long philtrum, wide nostril Fundus: diffuse optic disc pallor in both eyes ERG: mild retinal dysfunction	Depressed nasal bridge, wide nostrils, mid face hypoplasia	Broad forehead, depressed nasal bridge, long philtrum
Acrocephaly	+	-	+	+	+	+
Proptosis	+	+	+	+	+	+
Craniosynostosis	+	+	+	+	+	+
Frontal bossing	+	+	+	+	+	+
Congenital heart Disease	-	-	-	PDA, PFO	-	PFO
Syndactyly	Hands and feet	Hands and feet	Hands and feet	Hands and feet	Hands and feet	Hands and feet
Corpus callosum agenesis	-	-	-	-	+	-
Skeletal anomaly	Broad hands and toes	Broad hands and toes	Bilateral talipes equinovarus	-	Broad hands and feet	Broad hands and feet
Polydactyly	+	-	-	-	-	-
Oral anomalies Cleft palate Bifid uvula Impacted teeth Delayed eruption supernumerary teeth Thick gingiva	+ + - - - -	- + + - - -	+ - - - + -	+ + + - - _	- - - + - +	+ - - + - -
Genitalia	_	Right side hydrocele	-	-	-	-
Molecular analysis	*FGFR2*: (NM- 0001414- c.758 C > G p.(Pro253 Arg)	*FGFR2*: (NM- 0001414- c.758 C > G p.(Pro253 Arg)	*FGFR2*: (NM- 0001414- c.758 C > G p.(Pro253 Arg)	*FGFR2*: (NM- 0001414- c.758 C > G p.(Pro253 Arg)	*FGFR2*: (NM- 0001414- c.758 C > G p.(Pro253 Arg)	*FGFR2*: (NM- 0001414- c.758 C > G p.(Pro253 Arg)

*ERG* electroretinogram, *PDA* patente ductus arteriosus, *PFO* patent foramen ovale

## Molecular Results

**Fig. 2:**
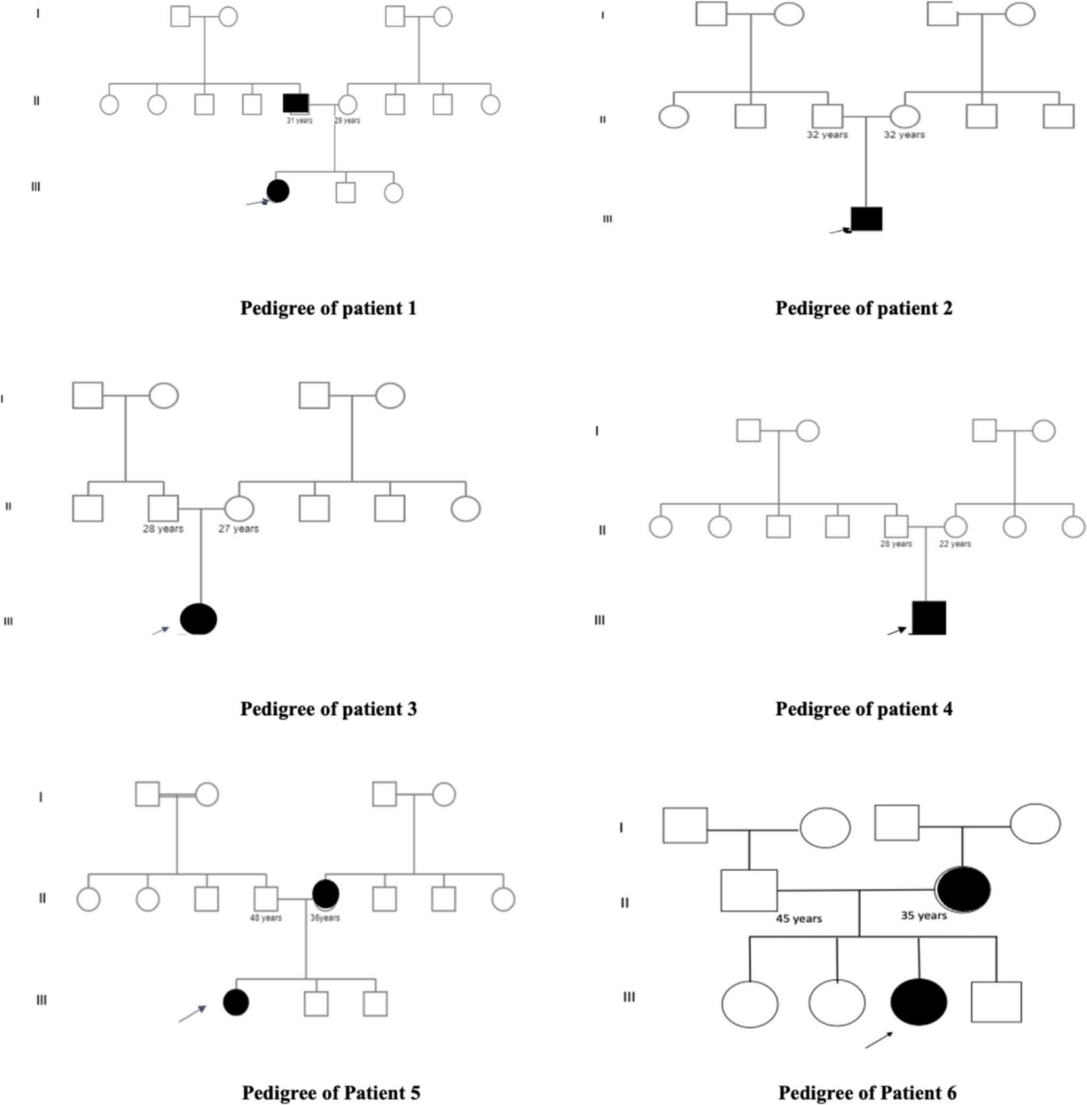
Three generations pedigrees of the six studied patients

**Fig. 3:**
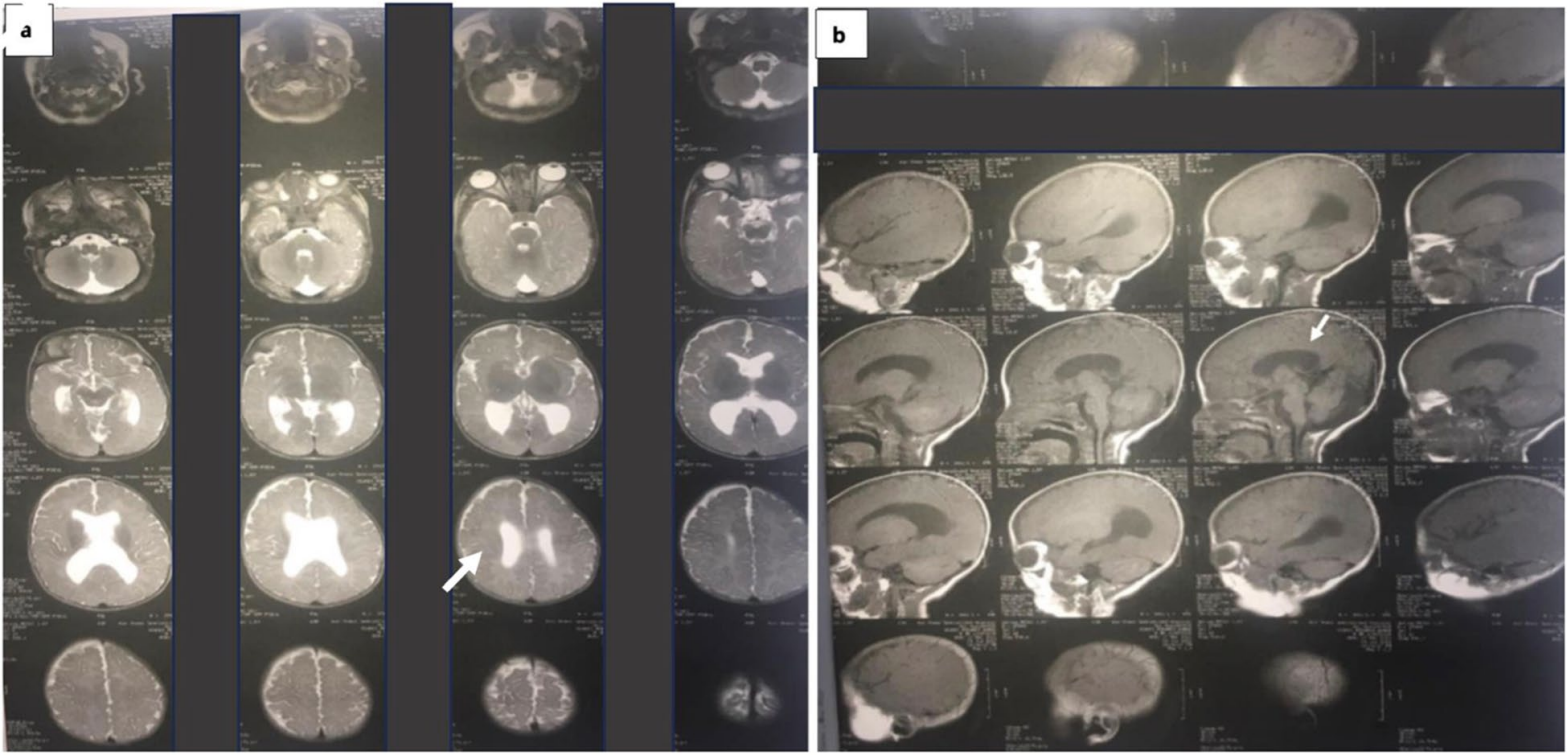
Brain MRI of patient 5 displayed agenesis of the corpus callosum. **a**. Axial T1-weighted brain MRI shows parallel lateral ventricles. **b**. The Sagittal T1-weighted MRI shows agenesis of the corpus callosum

Molecular analysis of all studied families provided valuable diagnostic data for AS, revealing the presence of the c.758 C > G (p.Pro253 Arg) variant in every patient examined. All patients were heterozygous for the reported variant following the autosomal dominant inheritance pattern. Moreover, segregation of the pathogenic variant within the families revealed three of the patients had parental inherited pathogenic variants Figs. 2 and 3.

**Fig. 4:**
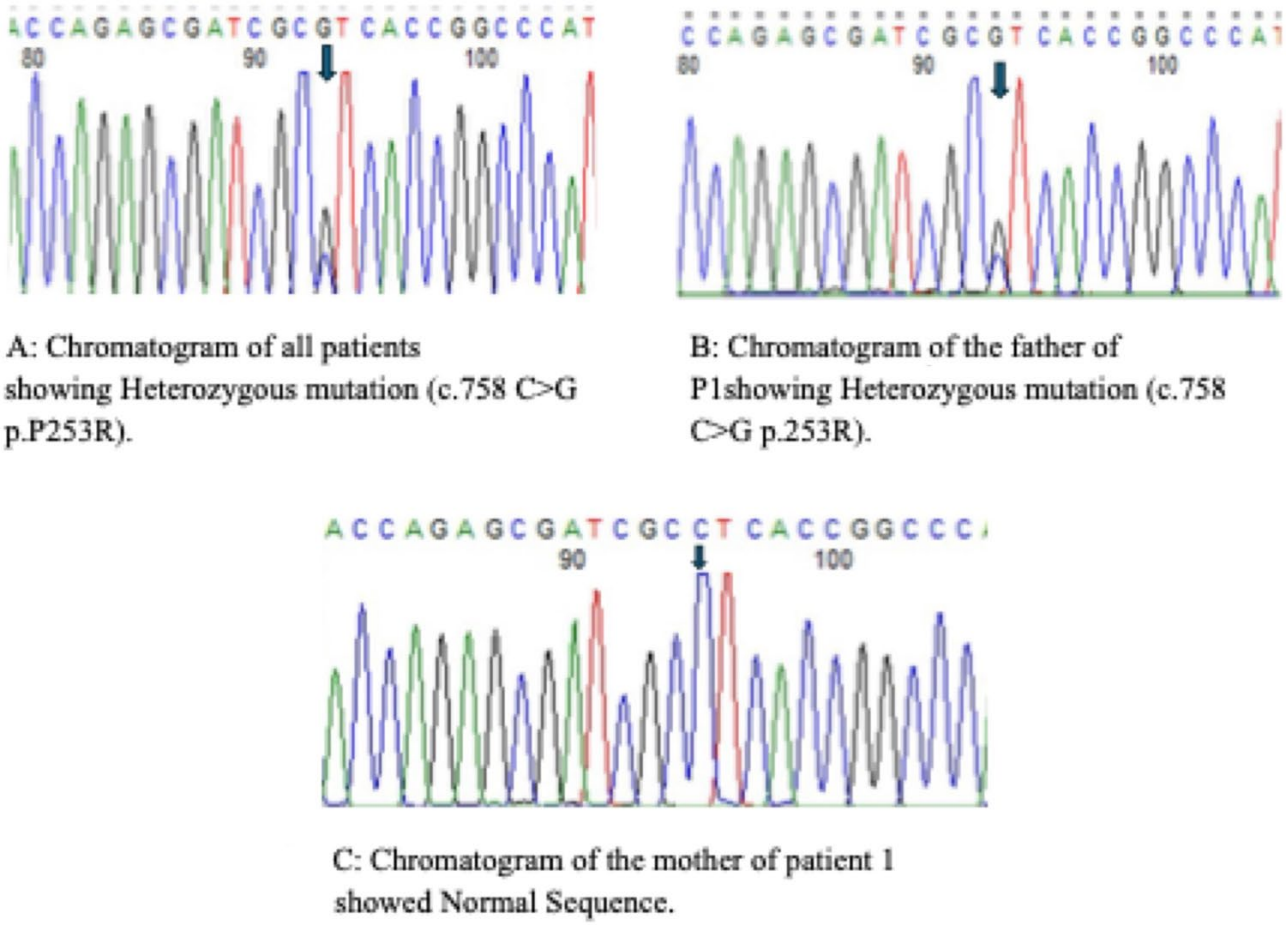
Chromatogram of the mother of patient 1 showed Normal Sequence

The identified causative pathogenic variant within the *FGFR2* gene has been previously reported in the Human Genome Pathogenic Variant Database (HGMD) (Fig. 4). In silico analysis predicted the variant’s pathogenicity (Table 2).

**Table 2:**
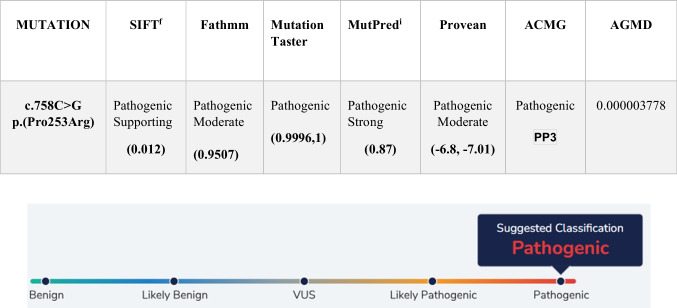
In silico analysis and the mutation prediction pathogenicity score for c.758 C>G p.(Pro253 Arg) in *FGFR2* gene

**Fig. 5:**
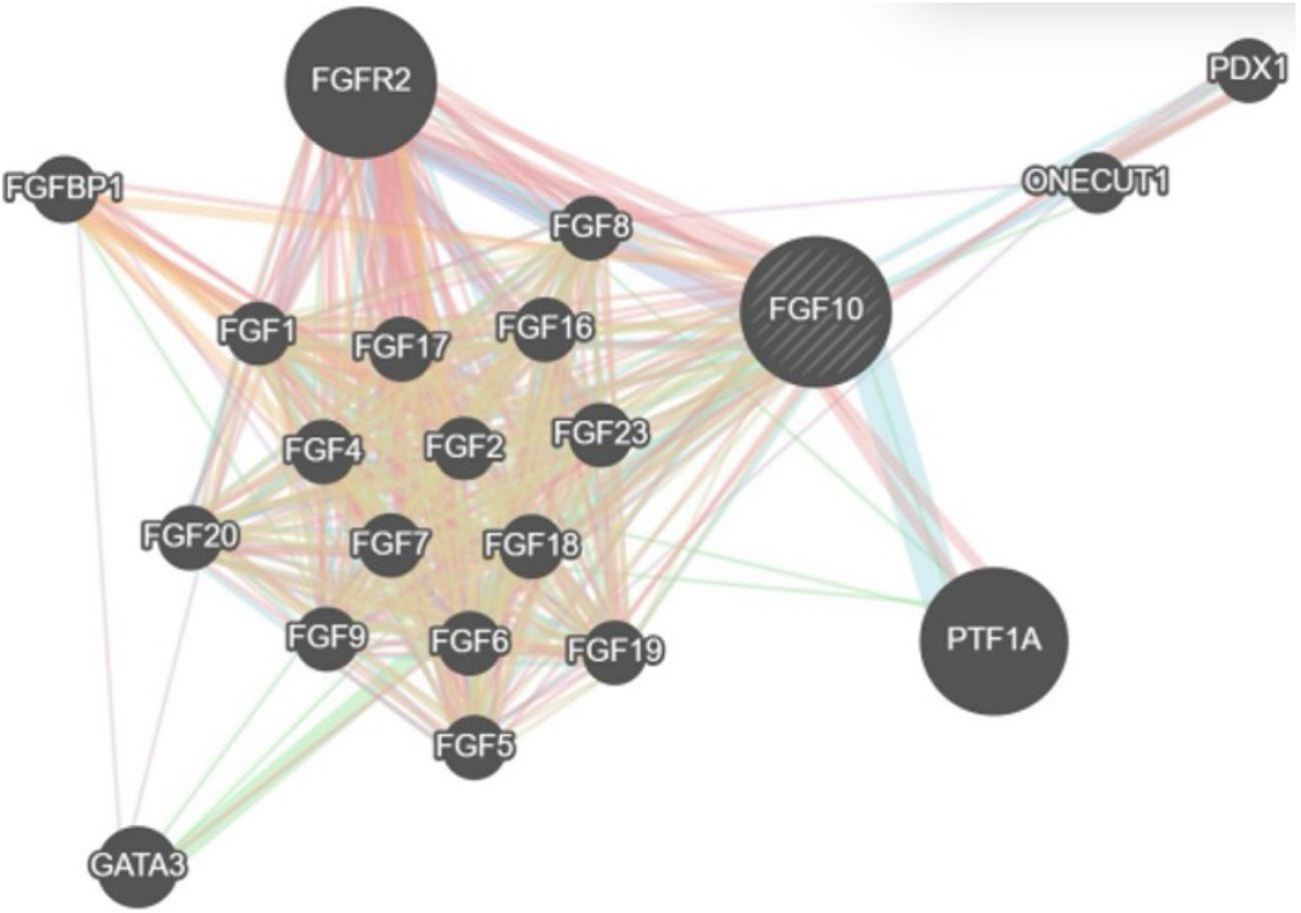
Shows the network produced by GeneMANIA of the *FGFR2* gene

The GeneMANIA results provided useful information on proteins and genes involved in physical interactions (77.64% and 8.01% coexpression), predicted sets of protein–protein interactions (5.37%), colocalization (3.63%), genetic interactions (2.87%), pathways (1.88%), and shared protein domains (0.60%). It also identified the other 20 genes interacting with *FGFR2* (Fig. 5).

## Discussion

This report highlights a significant breakthrough by presenting the largest cohort of Apert Syndrome (AS) cases among Egyptian patients. It includes six individuals diagnosed between the ages of 3 and 7 years. While previous research by Conrady et al. [6] reported an equal distribution of gender, our findings indicate a predominance of females, with four girls and two boys, yielding a ratio of 2:1. Importantly, all patients come from non-consanguineous marriages and have no family history of similar cases (see Fig. 2). Apert Syndrome is an autosomal dominant disorder, meaning there is a 50% chance that children will inherit the condition if one parent is affected. The presence of de novo mutations underscores that a lack of family history should not dismiss the possibility of a diagnosis. Additionally, Apert Syndrome demonstrates full penetrance and varying expressivity, resulting in considerable phenotypic diversity within families. This variation emphasizes the need for increased awareness and understanding of this condition [6].

Although previous studies have noticed a higher occurrence in children with older fathers, this condition can occur in children born to parents of all ages [23]. This is inconsistent with our studied cohort, where the paternal ages ranged from 26 to 48 years.

Several genetic syndromes present with craniosynostosis, a gain of function variants of the *FGFR2* gene, mainly in the third Ig-like domain and adjacent linker regions, leading to multiple types of autosomal-dominant craniosynostoses, such as Apert syndrome, Crouzon syndrome, Pfeiffer syndrome, and Beare-Stevenson cutis gyrata syndrome [24]. Pathogenic variants in the *FGFR2* gene, particularly the Ser252 Trp and Pro253 Arg variants, play an important role in bone growth disruption resulting in craniosynostosis and syndactyly the key diagnostic indicators of AS that differentiate it from other craniosynostosis syndromes [18, 25]. In our study, all patients presented with craniosynostosis and characteristic dysmorphic features, including depressed nasal bridge, proptosis, midface hypoplasia, and syndactly of hands and feet. Acrocephaly and frontal bossing were present in 5 patients (83%). Previous investigators asserted that all their studied patients showed similar phenotypes [26, 27].

Among system affections in Apert syndrome are central nervous system abnormalities, including corpus callosum deficits and structural heart defects in about 10% of patients [28]. The most frequent cardiac anomalies are ventricular septal defects, overriding aorta, or patent ductus arteriosus; some children have been documented to have complicated congenital heart disease [29]. Similarly, in the current study, patient 3 (16%) had congenital heart disease in the form of patent ductus arteriosus and patent foramen oval, and the brain MRI study of patient 5 detected central nervous system affection in the form of agenesis of the corpus callosum.

Also, previous studies, including the current one, documented skeletal anomalies among AS patients, where broad hands and toes were present in all patients, and bilateral talipes equinovarus were found in patient 3 [30]. Patient 1 had polydactyly, which is infrequently noted in cases with Apert syndrome [31].

Although none of the patients exhibited sensorineural hearing loss, various studies indicate that children with AS have a higher prevalence of hearing loss than the general population. It is important to understand the unique *FGFR2* pathogenic variations of each patient, as this knowledge is crucial for tailoring their clinical management. For example, a specific variation may provide insights into the severity of craniosynostosis or the likelihood of associated abnormalities, which can influence the type and timing of necessary surgical procedures [32]. A multidisciplinary approach is crucial for effectively managing Apert syndrome. This includes a team of specialists, such as geneticists, pediatricians, neurosurgeons, orthopedic surgeons, and speech therapists. Early detection through genetic testing facilitates prompt intervention, which can significantly enhance outcomes. For example, early cranial surgery can prevent increased intracranial pressure and support normal brain development, while timely orthopedic interventions can improve hand function and mobility [24]. Additionally, regular monitoring and coordinated care can address complications like hearing loss, vision problems, and respiratory issues, ensuring comprehensive patient health management. Genetic counseling is also essential for families to comprehend the inheritance patterns, recurrence risks, and available prenatal diagnostic options [23].

Dental examination of the patient is highly recommended because oral anomalies are detected in AS patients. Pseudo-prognathism, dental crowding, and maxillary hypoplasia have been previously reported [33].

An alteration in the *FGFR2* gene (fibroblast growth factor receptor 2) located on chromosome 10 results in Apert syndrome. More than 98% of AS cases are caused by de novo variants in the *FGFR2* gene, which plays an important role in bone growth. It is typically caused by specific missense variants of the highly conserved linker region between IgII and IgIII domains of *FGFR2*, most often either NM_000141.5(F*GFR2*): c.755 C > G (p.Ser252 Trp) or c.758 C > G (p.Pro253 Arg) [32]. Apert syndrome (AS) is thought to be caused by c.755 C > G (p.Ser252 Trp) Ser252 Trp variant in the *FGFR2* gene, which results in a malformed skull and delayed skeletal development due to early union of the cleft palate and craniofacial sutures [16, 34]. Despite being more prevalent worldwide and present in more than 67% of AS patients, the c.755 C > G (p.Ser252 Trp) Ser252 Trp variation was not detected in our patients [32].

To emphasize the genomic implications of the molecular targets of *FGF/FGFR* signaling in the regulation of osteoblastogenesis, the current study used the GeneMANIA program to assess gene interactions. This revealed a network of 20 linkages between the *FGFR2* genes. Several studies have also demonstrated the critical role that *FGFRs* play in controlling vitamin D and phosphate levels and the significance of the interaction between *FGF/FGFR* signaling and other signaling pathways in the regulation of osteogenesis [35]. Furthermore, *FGFR* signaling may regulate the process of *FGF23* expression in bone, indicating that *FGFRs* play dual roles in the FGF- 23/Klotho pathway, which promotes vitamin D and phosphate balance [36]. Nonetheless, it has been determined that many de novo missense variants in *FGFR2* cause AS [37]; in our study, three patients had parental inherited variants.

A mouse model study was performed to clarify the evidence of c.755 C > G (p.Ser252 Trp) Ser252 Trp, indicating that p38 and Erk1/2 have distinct roles in chondrogenic differentiation. The entire process of endochondral ossification is influenced by the p38 signaling pathway [38]. Erk1/2 simultaneously encourages bone marrow mesenchymal stem cells to differentiate into chondrogenic tissue at a later stage. Activation of the p38 and Erk1/2 pathways is partially responsible for the FGF-induced growth arrest of chondrocytes, and mitogen-activated protein kinase (MAPK) acts as a negative regulator of chondrogenesis [39].

The c.755 C > G (p.Ser252 Trp) Ser252 Trp pathogenic variant in the *FGFR2* gene was characterized in a previous report of an African American Apert syndrome female patient 37 years old with abnormal characteristics of the skull, face, and extremities that were detected at birth [40]. The patient had a c.755 C > G (p.Ser252 Trp) Ser252 Trp pathogenic variant in *FGFR2*. Another study also reported a young girl with early-onset low-grade papillary cancer and a c.758 C > G (p.Pro253 Arg) Pro253 Arg *FGFR2* pathogenic variant associated with Apert syndrome [41]. Both variants affect the highly conserved linker region between the immunoglobulin-like II and III domains, resulting in increased affinity and altered specificity of fibroblast growth factor (FGF) ligand binding [24]. These specific pathogenic variants cause abnormal intramembranous ossification, leading to premature fusion of sutures and an altered endochondral ossification in patients with Apert syndrome. All affected cases manifested in a heterozygous state for the pathogenic variant; the mutant alleles, therefore, exhibit dominance, and all the variants map to two specific regions of *FGFR2* that are specific to the Ser-Pro motif found at the 5′ end of the IgIIIa exon 7 [16]. A previous study provided evidence that suggests that *FGFR2* variants result in greater numbers of precursor cells entering the osteogenic pathway, which leads to premature calvarial ossification and bone matrix formation [41]. *FGFR2* variants may result in premature suture fusion through upregulation of epidermal growth factor (EGF) and platelet-derived growth factor (PDGF) α signaling [17]. The most important impact for genetic counseling in families with a history of affected AS patients is the noninvasive cell-free fetal DNA present in the maternal blood or using an amniotic fluid sample to assess for known pathogenic *FGFR2* gene variants in the first trimester in families with high-risk family histories of craniosynostosis. Limitations: Apert syndrome is a rare disorder; a limitation of this study is the small sample size. Future research should be conducted on a larger scale to corroborate these findings.

Conclusion: Our research presents the largest cohort of Apert syndrome in Egyptian patients, adding six new cases to the literature. This study highlights critical phenotypic characteristics and establishes that every patient possesses a pathogenic variant in the FGFR2 gene, underscoring the importance of genetic testing for accurate diagnosis and treatment. Raising awareness among healthcare providers is crucial for early diagnosis and timely intervention. Precise diagnosis and genetic counseling are essential for improving survival and preventing recurrence.

## Data Availability

No datasets were generated or analysed during the current study.
